# Role of Flaxseed Gum and Whey Protein Microparticles in Formulating Low-Fat Model Mayonnaises

**DOI:** 10.3390/foods11030282

**Published:** 2022-01-21

**Authors:** Keying Yang, Ruoting Xu, Xiyu Xu, Qing Guo

**Affiliations:** College of Food Science and Nutritional Engineering, China Agricultural University, Beijing 100083, China; s20203061042@cau.edu.cn (K.Y.); xuruoting1996@163.com (R.X.); xuxiyu2021@cau.edu.cn (X.X.)

**Keywords:** low-fat mayonnaise, whey protein microparticles, flaxseed gum, texture, rheology

## Abstract

Flaxseed gum (FG) and whey protein microparticles (WPMs) were used to substitute fats in model mayonnaises. WPMs were prepared by grinding the heat-set whey protein gel containing 10 mM CaCl_2_ into small particles (10–20 µm). Then, 3 × 4 low-fat model mayonnaises were prepared by varying FG (0.3, 0.6, 0.9 wt%) and WPM (0, 8, 16, 24 wt%) concentrations. The effect of the addition of FG and WPMs on rheology, instrumental texture and sensory texture and their correlations were investigated. The results showed that all samples exhibited shear thinning behavior and ‘weak gel’ properties. Although both FG and WPMs enhanced rheological (e.g., viscosity and storage modulus) and textural properties (e.g., hardness, consistency, adhesiveness, cohesiveness) and kinetic stability, this enhancement was dominated by FG. FG and WPMs affected bulk properties through different mechanisms, (i.e., active filler and entangled polysaccharide networks). Panellists evaluated sensory texture in three stages: extra-oral, intra-oral and after-feel. Likewise, FG dominated sensory texture of model mayonnaises. With increasing FG concentration, sensory scores for creaminess and mouth-coating increased, whereas those of firmness, fluidity and spreadability decreased. Creaminess had a linear negative correlation with firmness, fluidity and spreadability (R^2^ > 0.985), while it had a linear positive correlation with mouth-coating (R^2^ > 0.97). A linear positive correlation (R^2^ > 0.975) was established between creaminess and viscosity at different shear rates/instrumental texture parameters. This study highlights the synergistic role of FG and WPMs in developing low-fat mayonnaises.

## 1. Introduction

Dietary fats significantly contribute to the total energy and intake of lipophilic nutrients and bioactives. However, their excessive intake is an important factor leading to obesity and overweight [1], which has been linked to an increased rate of chronic diseases such as type-2 diabetes, cardiovascular diseases and certain cancers. The reduction in intake of fats is a generally recommended, non-invasive prevention strategy to combat obesity and overweight. However, food cravings and easily accessible, palatable lipid-based foods have made it hard to control their intake levels in certain groups of people.

Mayonnaise is one of the most popular sauces or condiments in the world. It is a mixture of egg yolk, vinegar, oil and spices and typically consists of 70–80% fat [2]. To meet an increasing consumer demand for healthier food products, developing low-fat mayonnaise without sacrificing food texture has emerged as an important task for researchers [2,3,4,5,6]. From the perspective of material science, traditional mayonnaise is a conventional oil-in-water (O/W) emulsion or a high internal phase O/W emulsion (>74% fat) [7]. At high oil concentrations, oil droplets are tightly packed together and become distorted from the spherical shape. The close packing of the droplets allows them to interact with one another and to form the gel-like structures, which imparts mayonnaise its main texture and mouthfeel, e.g., creaminess, thickness and smoothness [8,9,10,11,12,13,14]. Oil volume fraction plays a critical role in determining the sensory properties of liquid or semi-solid foods [15]. Oil droplets can effectively reduce friction because of the formation of a fat film following a plate-out mechanism [16]. Reduced friction is highly related to oil-related sensory properties and lubrication properties [14,17,18].

Fat substitution is a traditional approach for fat reduction and provides enhanced sensory properties and higher acceptability of the reduced-fat food products. In the development of these products, researchers employ biopolymers and their complexes to substitute fats in the food. Fat replacers can be categorized into two groups: (1) enhancing lubrication properties; (2) controlling bulk rheology. There have been many efforts to understand the lubrication properties of protein- or polysaccharide-based particles. Tribology research on the food emulsion system has revealed that the bio-based microspheres (e.g., whey protein microparticles) can effectively reduce the friction coefficient between the contacting surfaces via the ball-bearing mechanism, depending on their mechanical properties, surface properties, size and the volume fraction [19,20,21,22]. Furthermore, microparticles have successfully simulated sensory attributes of some emulsion-based foods. For example, mayonnaises substituted with the low-methoxyl pectin-based fat mimetics have the similar texture scores as those of full-fat mayonnaise [23]. Whey protein microparticles contribute to creaminess perception of the O/W emulsion due to their lubrication properties [20]. Furthermore, the addition of some biopolymers (e.g., xanthan, gellan and pectin) can significantly influence lubrication properties due to their adsorption onto the surfaces (i.e., forming film) and viscosity effect, subsequently improving lubrication-related sensory properties [24,25,26,27,28]. On the other hand, the bulk properties (e.g., viscosity) are the most important factor, determining many sensory properties of foods, such as firmness, adhesiveness, thickness and creaminess [29]. For example, the extensional viscosity is correlated to a slimy, sticky and mouth-coating perception in xanthan gum solutions; the instrumental viscosity of bovine milk is positively correlated with the sensory viscosity; the thickness perception is correlated to the shear viscosity of thick solutions prepared by xanthan and dextran [30,31,32]. In practice, biopolymers with high functionalities (e.g., whey proteins, pectin and chia seed mucilage) have been successfully used to replace fats through enhancing bulk properties in different liquid or semi-solid food systems, such as mayonnaises and yogurts [33,34,35,36,37]. However, there is still a lack of a profound understanding of the relationship between bulk rheology at different deformations and sensory properties, which is critical to the development of a low-fat mayonnaise with high sensory acceptability. In particular, the respective role of bio-based microspheres and thickening agents in the bulk rheology and sensory properties of mayonnaises warrants further study.

In this study, we reformulated model mayonnaises using traditional ingredients (e.g., dry egg yolk, corn oil, vinegar, sucrose, salt) and substituted fats using whey protein microparticles (WPMs) of 10–20 µm and flaxseed gum (FG) (i.e., food reformulation), aiming to understand the underlying mechanisms of the effect of the addition of FG and WPMs on bulk properties (i.e., rheology and instrumental texture). Moreover, we investigated the sensory texture of model mayonnaises, including creaminess and creaminess-related textural attributes, aiming to clarify how the addition of FG and WPMs changed the creaminess.

## 2. Materials and Methods

### 2.1. Materials

Whey protein isolate (WPI) (Bipro, >91% protein) was purchased from Davisco Foods International (Eden Prairie, MN, USA). CaCl_2_ (≥97%) and NaCl (≥99%) were purchased from Sigma-Aldrich Merck KGaA (Darmstadt, Germany). FG was purchased from Linseed Biologic Technologies Co., Ltd. (Xinjiang, China) and used without further purification. Nile Red and Fast Green were obtained from Solarbio (Beijing, China). Sucrose, salt, corn oil, dried egg yolk, whole milk, whipped cream, jam, mayonnaise, vinaigrette and vinegar were purchased from a local supermarket (Beijing, China). Double-distilled water was used to prepare all solutions and mayonnaises. All other reagents were obtained from Sinopharm Chemical Reagent Co., Ltd. (Shanghai, China) and were of analytical grade.

### 2.2. Preparation of Whey Protein Microparticles (WPMs)

A 10 wt% WPI solution was prepared by dissolving 100 g WPI into 900 g water. WPI solutions were stored at 4 °C overnight to allow complete hydration. Then, 10, 15, 20, 25, 35 or 50 mM CaCl_2_ was added into the WPI solutions. Whey protein gels containing different CaCl_2_ concentrations were formed by heating the WPI solutions at 90 °C for 30 min and cooling at room temperature. WPMs were prepared by (1) crushing whey protein gels to smaller pieces; (2) adding water into gel pieces with a ratio of 1:9; (3) homogenizing gel pieces at 9000, 12,000, 15,000, 18,000 or 21,000 rpm for 4 min using an Ultra Turrax (S18N-19G, IKA, KG, Staufen, Germany) at room temperature.

### 2.3. Scanning Electron Microscopy (SEM)

Whey protein gels were vacuum freeze-dried at −83 °C for 48 h. The freeze-dried gels were sputtered with a platinum conductive layer. The microstructure of different samples was captured using a SEM (UHR FE-SEM SU8200 Series, Hitachi, Kyoto, Japan) at an accelerating voltage of 3 kV.

### 2.4. Particle Size Measurement

The particle size distribution of WPMs was measured using a Particle Size Analyzer (LS 13 320, Beckman Coulter, Indianapolis, IN, USA). The refractive index of water and WPI was 1.33 and 1.48, respectively. The particle size was reported as the volume-weighted mean diameter (D_4,3_).

### 2.5. Mechanical Properties of Whey Protein Gels

The mechanical properties of the gels containing different CaCl_2_ concentrations were determined using a texture analyzer (TA.XT2, Stable Micro System Ltd., Haslemere, Surrey, UK). Cylinder samples (20 mm in height and 25 mm in diameter) were compressed between two flat plates up to 30% strain with the test speed of 1 mm s^−1^ and trigger force of 0.1 N. The force at 30% strain was defined as gel hardness. Young’s moduli were calculated from the linear regime of the force-strain curves (<5%). The penetration test was performed to a strain of 80% with a probe (6 mm in diameter). The test speed was 1 mm s^−1^, with a trigger force of 0.08 N. The fracture force and strain of the gels were recorded.

### 2.6. Swelling Test

To mimic the physicochemical environment of model mayonnaises, the swelling medium was prepared by dissolving 5 wt% sugar and 0.5 wt% NaCl into water, and the pH was adjusted to 4. Cylinder gels (25 mm in diameter and 10 mm in height) were weighed (w_0_) and then immersed in the swelling medium for 0, 3, 8, 16, 24, 30 and 54 h. After static soaking, the samples were weighed again (W_n_). The swelling ratio was calculated by the following equation:

(1) $$\mathrm{Swelling}\;\mathrm{ratio}\;\left(\%\right)=\frac{w_{n}-w_{0}}{w_{0}}\times 100\%$$

### 2.7. Preparation of Model Mayonnaises

The ingredients of the model mayonnaises are listed in Table 1. FG and sugar were added into hot water and stirred at 80 °C for 30 min using a magnetic mixer (C-MAG HS 7, IKA, KG, Staufen, Germany) (mixture 1). WPMs, salt and dried egg yolk were added into water, followed by a homogenization at 10,000 rpm for 20 s using an Ultra Turrax (S18N-19G, IKA, KG, Staufen, Germany). Then, corn oil was added and homogenized at 10,000 rpm for 20 s (mixture 2). Finally, model mayonnaises were obtained by blending vinegar, mixture 1 and mixture 2.

### 2.8. Rheological Measurement

The rheological properties of model mayonnaises were investigated using a stress-controlled rheometer (DHR-2, TA Instruments, New Castle, DE, USA) with a serrated parallel plate geometry (40 mm in diameter) and 800 μm gap. A shear rate range of 0.001–1000 s^−1^ at a constant temperature of 25 °C was used to determine the steady shear flow behavior of the samples. Dynamic oscillatory shear testing was performed by means of a strain amplitude sweep (1 Hz, 0.1–50% strain) and a frequency sweep in the linear viscoelastic region (1% strain, 10–0.1 rad/s). The ability of the samples to resist the deformation under the applied strain was recorded as the storage modulus (G′), loss modulus (G″) and phase angle (δ). All experiments were carried out at least in triplicate.

### 2.9. Instrumental Texture Measurement

The instrumental texture of model mayonnaises was measured by a texture analyzer (TA Instruments, New Castle, DE, USA) with a cylinder probe (36 mm in diameter). Each sample was filled to a 50 mm depth in a beaker with dimensions of 40 mm in diameter and 60 mm in height. The samples were then penetrated to a depth of 30 mm at a speed of 1.5 mm s^−1^ with a trigger force of 3 g. Textural parameters of the samples (i.e., peak force, positive area, negative peak force, negative area) were calculated. All experiments were carried out at least in triplicate.

### 2.10. Confocal Laser Scanning Microscopy

Microstructure of model mayonnaises was observed using a confocal laser scanning microscope (LSM710, Zeiss, Germany). The oil and protein phases of the samples were stained by Nile Red solution (0.1 wt% in acetone) and Fast Green solution (0.2 wt%), respectively. A small drop of the sample was placed on a concave confocal microscope slide, mixed with 10 mL Nile Red and 10 mL Fast Green, stained for 30 min and then covered with a cover slip. A 488 nm argon laser and a 633 nm He–Ne laser were used to excite the oil and protein phases, respectively. Emission spectra above 505 nm for the oil phase and above 650 nm for the protein phase were collected. It should be noted that model mayonnaises with a high concentration of FG and WPMs were hard to stain. Therefore, high-quality images were acquired for the samples containing 0.3 wt% FG or 0.6 wt% FG + 0 wt% WPMs in this study.

### 2.11. Stability Test

Fresh model mayonnaises were stored at 25 °C in a glass tube and monitored by a digital camera over 27 days (1, 7, 18, 27 d) to test their storage stability. Model mayonnaises were put into 5 mL test tubes that were sealed with plastic caps and then centrifuged at 4000 rpm for 8 min and at 10,000 rpm for 10 min using a high-speed centrifuge (TG16-WS, Cence, Changsha, China) to test their centrifugation stability.

### 2.12. Sensory Evaluation

Panellists aged between 18 and 30 years old were recruited and selected based on strict dental criteria as well as their basic texture, taste and flavor detection. Sensory tests were carried out in compliance with the human ethics committee at China Agricultural University. 

Training was executed in 4 sessions of 1 h each with 5 common commercial semi-solid foods (i.e., whole milk, whipped cream, jam, mayonnaise, vinaigrette). An attribute list containing preliminary attributes (extra-oral, intra-oral and after-feel) with precise definitions and rating instructions was provided to panellists during the attribute generation by the panel (Table 2). Each panelist was offered a plastic spoon and 20 g of each commercial semi-solid food. Panelists were instructed to rate extra-oral attributes before ingestion of food and intra-oral attributes with the tongue and palate. After-feel attributes were rated after swallowing. The attribute generation and definition were completed after 2 sessions, followed by one more session to reach consensus between panellists on rating the attributes. In the last training session, panellists were asked to rate 5 commercial samples and discuss the results.

A total of 12 model mayonnaise samples, labeled with random three-digit numbers, were evaluated in individual sensory booths at 25 °C under normal light conditions during 3 sessions of 1 h each. All model mayonnaise samples were prepared 12 h before sensory testing and served in a randomized order to panellists. The panellists rated the mayonnaise samples using a 100 mm continuous line anchored with ‘very little’ at 0% and ‘very much’ at 100% of the line scale, on which participants marked an ‘X’. Scores were determined by the distance (in mm) from the left starting point of the line to the ‘X’. All panellists followed the same evaluation procedure. First, they were instructed to assess textural (extra-oral) attributes, then to eat the samples once to evaluate textural (intra-oral) attributes. Finally, after-feel attributes were evaluated after swallowing. Panellists rinsed and cleaned their mouth with water before evaluating the next sample.

### 2.13. Statistical Analysis

All experiments were performed at least in triplicate. The results were expressed as means with standard deviations and were analyzed by SPSS version 25 (SPSS Inc., Chicago, IL, USA). A one-way analysis of variance (ANOVA) and Fisher least significant difference test at *p*  <  0.05 were used to determine significant differences between means. A Pearson correlation test was performed to explore the correlation between creaminess and other sensory texture attributes/rheological parameters/instrumental texture parameters at *p* < 0.05 or 0.01. Data were normalized for all correlation tests.

## 3. Results and Discussion

### 3.1. Whey Protein Microparticle Properties

**Figure 1:**
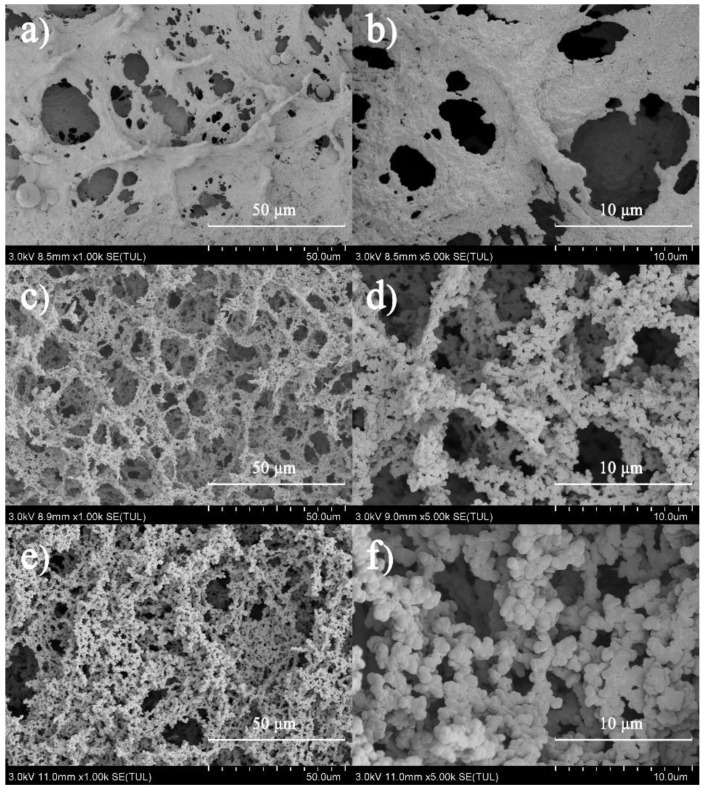
Scanning electron micrographs of the heat-set whey protein gels containing different CaCl_2_ concentrations with different magnifications. (**a**) 10 mM CaCl_2_, 1000×; (**b**) 10 mM CaCl_2_, 5000×; (**c**) 15 mM CaCl_2_, 1000×; (**d**) 15 mM CaCl_2_, 5000×; (**e**) 50 mM CaCl_2_, 1000×; (**f**) 50 mM CaCl_2_, 5000×.

In this study, the properties of whey protein microparticles were represented by those of the heat-set whey protein gels (Figure S1). Figure 1 shows SEM images of the heat-set whey protein gels containing different CaCl_2_ concentrations. A relatively uniform film-like network structure was observed at low CaCl_2_ concentration (Figure 1a,b). As CaCl_2_ concentration increased to 15 mM, denser and larger protein aggregates were formed with significant phase separation, which assembled into the 3-dimensional (3D) gel network (Figure 1c,d). With further addition of Ca^2+^ (50 mM), the protein molecules aggregated into the spherical microgels of several microns, which formed a particulate gel network. This evolution of the gel structure was mainly ascribed to electrostatic shielding by Ca^2+^, which could lower repulsion between protein molecules and promote their interactions [38,39].

**Figure 2:**
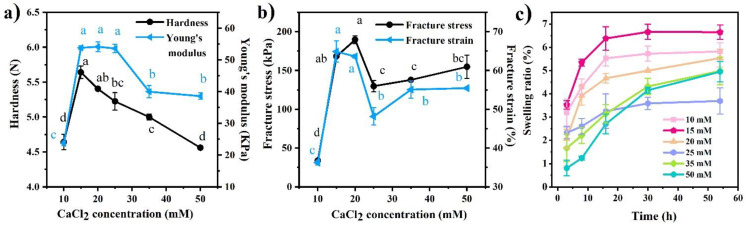
(**a**) Hardness and Young’s modulus, (**b**) fracture stress and strain and (**c**) swelling ratios of the heat-set whey protein gels containing different CaCl_2_ concentrations. Different letters in the same curve indicate significant difference at *p* < 0.05.

The effect of CaCl_2_ concentration on the large deformation properties of the whey protein gels is illustrated in Figure 2a. Hardness and Young’s modulus reached the highest value at 15 mM. With further addition of CaCl_2_, hardness gradually decreased; Young’s modulus did not change significantly until CaCl_2_ concentration reached 25 mM. By contrast, the fracture stress and strain peaked at 15–20 mM, significantly decreased at 25 mM, and plateaued with further addition of CaCl_2_ (Figure 2b). In theory, the magnitude of interactions between protein molecules per unit volume of the gel increased with CaCl_2_ concentration [39,40]. However, both large deformation and fracture properties did not correlate with CaCl_2_ concentration, indicating the important role of the gel structure in determining mechanical properties. As shown in Figure 1, a large volume of pores was generated at higher CaCl_2_ concentrations (>15 mM), which caused friction during the large deformation and subsequently reduced gel hardness [41,42]. The strength of the gel network itself plays a critical role in determining fracture mechanics, which has a highly positive correlation with CaCl_2_ concentration [39,43]. This could mediate the adverse effect of pores and cracks on fracture properties that were formed in the gels containing high CaCl_2_ concentrations [44].

The swelling ability of the heat-set whey protein gels is shown in Figure 2. All gels swelled rapidly during the first 8 h of soaking, and then their swelling slowed down. In general, the swelling ratio of the gels containing higher CaCl_2_ concentrations (25, 35, 50 mM) was larger than that of those containing lower CaCl_2_ concentrations (10, 15, 20 mM). However, the swelling ratio of all gels was lower than 7% over 54 h of soaking, which could be ascribed to the acidic pH near to the isoelectric point of WPI and high ionic strength of the environmental solution [45,46]. 

WPMs were prepared by mechanically breaking the whey protein gels. The D_4,3_ of WPMs containing 10 mM CaCl_2_ decreased from 40 to 10 µm with increasing homogenization speed from 9000 to 21,000 rpm because of increased energy input (Table S1). By contrast, the D_4,3_ of WPMs containing 15 mM CaCl_2_ became much larger at the same speed because of higher gel strength (Table S1). Therefore, the soft whey protein gel containing 10 mM CaCl_2_ was selected to produce WPMs of <20 µm in this study.

### 3.2. Rheology and Instrumental Texture of Model Mayonnaises

**Figure 3:**
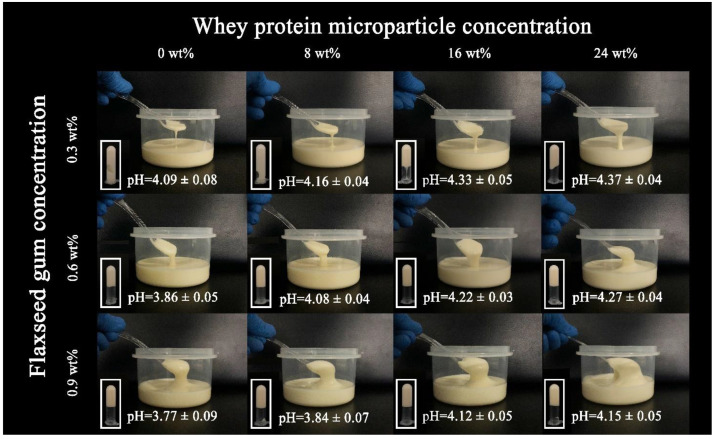
Photographs of model mayonnaises containing different concentrations of the flaxseed gum and whey protein microparticles.

A series of 3 × 4 fresh model mayonnaises were prepared by varying FG weight/WPM weight ratios (Figure 3). With increasing FG and WPM concentration, model mayonnaises visually became more viscous; the addition of FG affected the viscosity of model mayonnaises more significantly.

**Figure 4:**
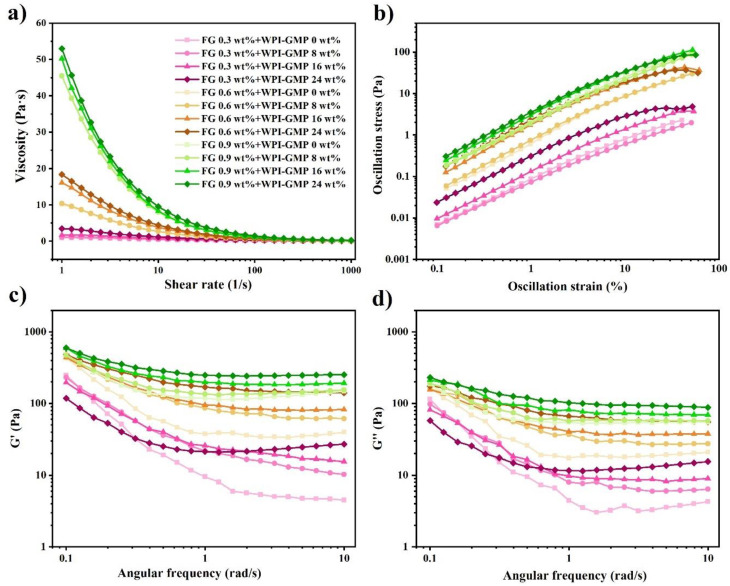
(**a**) Viscosity vs. shear rate flow curves of model mayonnaises, (**b**) strain amplitude sweep of model mayonnaises in the oscillatory shear test, (**c**,**d**) frequency sweep of model mayonnaises in the oscillatory shear test.

The viscosity vs. shear rate of model mayonnaises is shown in Figure 4a. Shear thinning occurred in all samples. The thickening effect of WPMs was not significant at low concentration (8 wt%), suggesting that the interparticle interactions and the particle-continuous phase interactions were relatively weak. By contrast, the addition of FG could effectively enhance flow resistance at a relatively low concentration (0.6 wt%), which was ascribed to entangled polysaccharide chains and their interactions with other molecules [47,48]. In this regard, FG played a more important role in enhancing the viscosity of model mayonnaises.

Model mayonnaises had a large linear viscoelastic region up to 10% strain. Although the addition of both FG and WPMs caused a higher yield stress, the former had a relatively larger effect. Both yield stress and strain increased with the addition of FG, whereas the yield stress and strain increased and decreased with the addition of WPMs, respectively, indicating that FG and WPMs influenced the structure of mayonnaises through different mechanisms [49]. WPMs probably acted as an active filler to strengthen the structural network of the samples. However, the slip between WPMs and the continuous phase might occur under the high strain, leading to reduced yield strain [50]. On the other hand, entangled linear FG macromolecules could bear a larger strain due to the formation of a 3D network [51]. 

Oscillatory shear testing was conducted at the linear viscoelastic region. During the frequency sweep, the storage moduli of all samples were higher than the loss moduli, indicating that solid-like properties dominated in the model mayonnaises. The frequency sweep was conducted from the high to low frequency. At the high frequency, the samples had lower storage and loss moduli, suggesting that the structure of the samples was partially disrupted [52]. With the decrease of the frequency, the storage and loss moduli gradually increased, indicating that this structural disruption was reversible. Moreover, the addition of both FG and WPMs led to a lower phase angle (i.e., a higher elasticity) (Figure S2), suggesting that the structure of the mayonnaise was enhanced by the addition of FG and WPMs.

**Figure 5:**
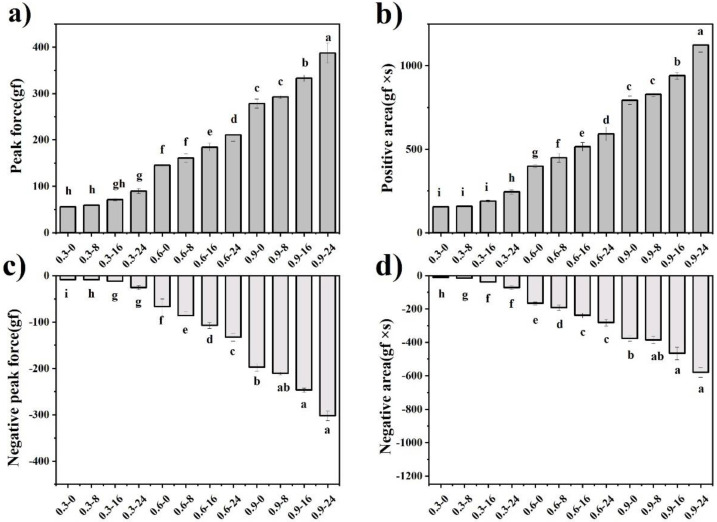
Instrumental texture parameters of model mayonnaises: (**a**) peak force-hardness; (**b**) positive area-consistency; (**c**) negative peak force-adhesiveness; (**d**) negative area-cohesiveness. Different letters above the bars indicate significant difference at *p* < 0.05.

The instrumental texture of model mayonnaises was measured using the back extrusion test. The peak force, positive area, negative peak force and negative area represent hardness, consistency, adhesiveness and cohesiveness, respectively. As shown in Figure 5, these instrumental textural parameters were enhanced by the addition of FG and WPMs. FG was capable of enhancing textural properties more effectively, which was similar to its effect on rheological properties. A low concentration of WPMs had a limited effect on textural properties. When WPM concentration reached 16 wt%, the enhanced effect became evident probably because of the interparticle interactions and the particle-continuous phase interactions, which was consistent with the results of rheological properties.

**Figure 6:**
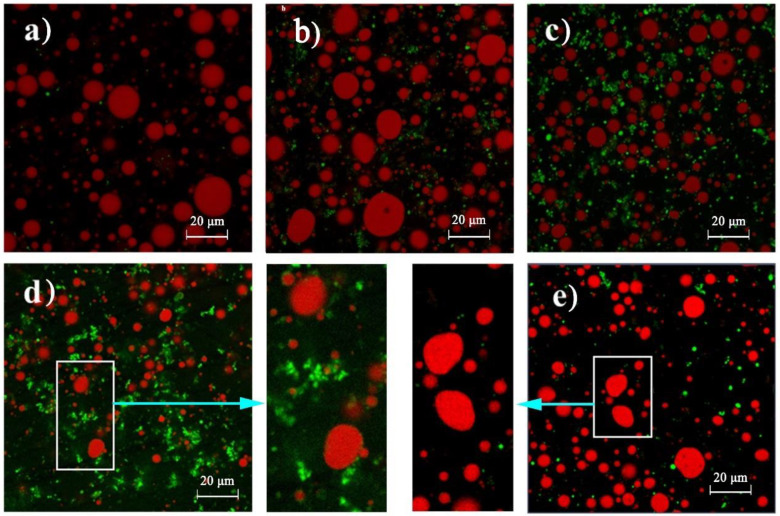
Confocal laser scanning microscopy images of model mayonnaises containing different concentrations of the flaxseed gum (FG) and whey protein microparticles (WPMs). (**a**) 0.3 wt% FG + 0 wt% WPMs, (**b**) 0.3 wt% FG + 8 wt% WPMs, (**c**) 0.3 wt% FG + 16 wt% WPMs, (**d**) 0.3 wt% FG + 24 wt% WPMs, (**e**) 0.6 wt% FG + 0 wt% WPMs.

The microstructure of model mayonnaises is shown in Figure 6. The oil droplet size of model mayonnaises was of several microns to ~20 microns. With the addition of WPMs, the oil droplet size decreased because of the emulsifying role of WPMs in emulsion formation, i.e., WPMs adsorbed on the oil-water interface (Figure 6a–d). At the high concentration of WPMs (16 and 24 wt%), the aggregation of WPMs occurred with the formation of the network (Figure 6d), which explained that WPMs could significantly enhance rheological and textual properties at the high concentration. Moreover, oil droplets were deformed into a non-spherical shape, although the oil volume fraction (15%) was much lower than 74%, suggesting that FG and WPMs could affect structural and rheological/mechanical properties through a mechanism similar to the compact packing of oil droplets. 

**Figure 7:**
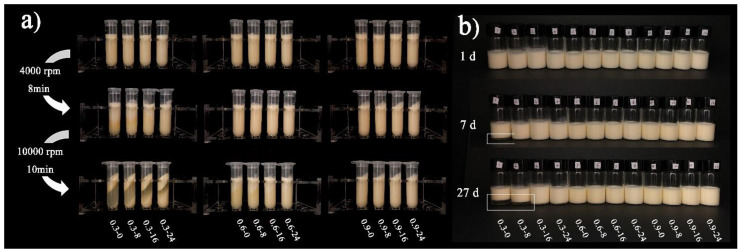
Kinetic stability of model mayonnaises: (**a**) centrifugation stability; (**b**) storage stability.

The centrifugation stability of model mayonnaises is illustrated in Figure 7a. After centrifugation at 4000 rpm, phase separation occurred in model mayonnaises containing 0.3 wt% FG; the samples containing 0.6 and 0.9 wt% FG remained stable. With increasing centrifugation speed to 8000 rpm, severe phase separation occurred in the 0.3 wt% FG samples. Slight phase separation occurred in the 0.6 wt% FG samples. With the addition of 0.9 wt% FG, the mayonnaises were stable. The stability of model mayonnaises during storage is shown in Figure 7b. Creaming occurred in the samples containing 0.3 wt% FG + 0 wt% or 8 wt% WPMs after 27 days of storage, whereas the samples containing 0.3 wt% + 16 or 24 wt% WPMs were stable. All other samples were relatively stable during storage. The role of FG and WPMs in stabilizing model mayonnaises could be ascribed to the increase of bulk viscosity [53,54]. Moreover, the reduction of oil droplet size induced by the addition of WPMs also contributed to the stability of model mayonnaises.

### 3.3. Sensory Texture of Model Mayonnaises

**Figure 8:**
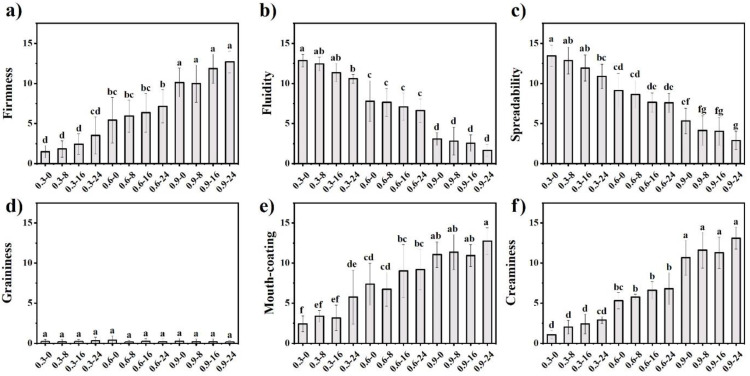
Sensory texture of low-fat model mayonnaises: (**a**) firmness, (**b**) fluidity, (**c**) spreadability, (**d**) graininess, (**e**) mouth-coating, (**f**) creaminess. Different letters above the bars indicate significant difference at *p* < 0.05.

Sensory evaluation of mayonnaise texture was conducted in three stages: (1) extra-oral; (2) intra-oral; (3) after-feel (Figure 8). Firmness perceived by stirring the samples increased significantly with FG concentration, whereas the effect of the addition of WPMs on firmness was not significant (Figure 8a). Fluidity in the mouth significantly decreased with FG concentration (Figure 8b). Interestingly, panellists could perceive the fluidity difference between 0.3 wt% FG samples containing different WPM concentrations. With increasing FG concentration, the fluidity difference generated by the addition of WPMs was neglected by panellists because of the much larger contribution of FG to fluidity. Spreadability significantly decreased with FG concentration (Figure 8c). The addition of WPMs also significantly reduced spreadability, regardless of FG concentration, suggesting that a direct relationship existed between spreadability and viscosity [55]. When model mayonnaises were spread into a thin film, panellists could hardly perceive graininess for all samples (Figure 8d), suggesting that the size and mechanical strength of WPMs were below the perceived threshold [56,57]. FG was the key factor generating perceived mouth-coating (Figure 8e). WPMs had a limited effect on perceived mouth-coating of model mayonnaises, although this effect was more significant at a low FG concentration (0.3 wt%).

Creaminess increased greatly with FG concentration (Figure 8f). Although there was no difference in statistics, creaminess increased with the addition of WPMs, which was in agreement with previous studies. For example, Liu et al. reported that microparticulated whey protein contributed to fat-related sensations (e.g., creaminess and fattiness) through enhancing lubrication properties [20]. Moreover, the addition of microparticulated whey protein significantly enhanced creaminess of low-fat yogurts, the effect of which depended on their particle size [58]. Creaminess represents an overall sensory texture, which is a combination of different textural perceptions [59,60]. As shown in Table 3, creaminess had a linear negative correlation with firmness, fluidity and spreadability, while it had a linear positive correlation with mouth-coating. This suggests that thickness perception (i.e., lower fluidity and spreadability) and sticky mouth-coating could be the key factors giving rise to creaminess. Meanwhile, this strongly supports that creaminess is a combination of complex textural attributes.

### 3.4. Correlation between Creaminess and Rheology/Instrumental Texture

Viscosities of the 12 samples at different shear rates were used to analyze their correlation with creaminess (Table 4). The results showed that creaminess had a linear positive correlation with the viscosity measured at different shear rates. Although the change in viscosity in the model mayonnaise systems was driven by different mechanisms (e.g., active fillers and entangled linear macromolecules), viscosity that represents the bulk properties could reflect the sensory creaminess of mayonnaises to a relatively high extent. This was in agreement with a previous study on protein beverages, with a key finding that the primary effect on viscosity-related sensory properties (e.g., creaminess, consistency and mouth-coating) was because of increased viscosity for a wide range of viscosity levels [61]. Strong correlation between viscosity and creaminess was also found in other emulsion-based foods, e.g., low-fat yogurts and custards [49,58]. Textural parameters measured by the texture analyzer using different methods (e.g., TPA and back extrusion) are widely used to predict the sensory properties of semisolid and solid foods [62,63]. There was a linear positive correlation between creaminess and four instrumental texture parameters (i.e., hardness, consistency, cohesiveness and adhesiveness), suggesting that instrumental texture measured at large deformations could reflect the creaminess of mayonnaises to some extent [24]. Recently, Conti-Silva et al. investigated the correlations between instrumental texture and oral viscosity using eight different food samples, i.e., water, condensed milk, strawberry yogurt, honey, creamy dairy dessert, UHT cream, petit suisse strawberry flavor, and dulce de leche [64]. The authors found that oral viscosity was positively correlated with positive areas from the curves obtained in the back extrusion test.

## 4. Conclusions

The addition of FG and WPMs enhanced the rheological and textural properties of model mayonnaises; this effect was largely attributed to the addition of FG, which probably formed an entangled 3D network. WPMs worked as active fillers and influenced rheological/textural properties through the interparticle interactions and the particle-continuous phase interactions at a relatively high concentration. We found that apparent viscosities measured at different shear rates and instrumental texture parameters measured by the back extrusion test had a linear positive correlation with creaminess. Moreover, creaminess was highly correlated to extra- and intra-oral textural properties, suggesting that creaminess in the context of texture is a reflection of overall textural perceptions. In particular, the thick perception (low fluidity and spreadability) and sticky mouth-coating were important factors giving rise to creaminess. In future studies, the synergistic effect of FG and WPMs on the tribology of low-fat mayonnaises and its correlation with textural perception will be investigated.

## Figures and Tables

**Table 1:** Ingredients of model mayonnaises.

Number of Sample	FG	WPM	Sucrose	Salt	Vinegar	Corn Oil	Dry Egg Yolk
	wt%	wt%	wt%	wt%	wt%	wt%	wt%
0.3–0	0.3	0	5	0.5	4	15	4
0.3–8	0.3	8	5	0.5	4	15	4
0.3–16	0.3	16	5	0.5	4	15	4
0.3–24	0.3	24	5	0.5	4	15	4
0.6–0	0.6	0	5	0.5	4	15	4
0.6–8	0.6	8	5	0.5	4	15	4
0.6–16	0.6	16	5	0.5	4	15	4
0.6–24	0.6	24	5	0.5	4	15	4
0.9–0	0.9	0	5	0.5	4	15	4
0.9–8	0.9	8	5	0.5	4	15	4
0.9–16	0.9	16	5	0.5	4	15	4
0.9–24	0.9	24	5	0.5	4	15	4

**Table 2:** Textural attributes and definitions.

Stages	Attributes	Definitions
Texture (extra-oral)	Firmness	Degree of resistance when stirring with a spoon
Texture (intra-oral)	Fluidity	Degree of the sample flowing on the tongue
	Spreadability	Degree of spreading the sample into a thin film by moving the tongue up and down against the palate
	Graininess	Extent of perception of particles when the tongue slides along the palate
	Mouth-coating	Degree of coating in the mouth after swallowing
After-feel	Creaminess	Degree to which the sample leaves a soft, velvety, fatty feeling after swallowing. Creaminess is perceived in the whole mouth.

**Table 3:** Correlation between creaminess and extra- and intra-oral textural attributes.

	Extra-Oral	Intra-Oral		
**Creaminess**	**Firmness**	**Fluidity**	**Spreadability**	**Mouth-Coating**
	−0.986 **	−0.994 **	−0.992 **	0.970 **

** indicates significant difference at *p* < 0.01.

**Table 4:** Correlation between creaminess and instrumental texture parameters/viscosities at different shear rates.

Shear Rate (s^−1^)	R^2^	Instrumental Texture	R^2^
1	0.977 **	Hardness	0.990 **
10	0.992 **	Consistency	0.988 **
100	0.992 **	Adhesiveness	0.985 **
1000	0.986 **	Cohesiveness	0.984 **

** indicates significant difference at *p* < 0.01.

## Data Availability

Not applicable.

## Supplementary Materials

The following supporting information can be downloaded at: https://www.mdpi.com/article/10.3390/foods11030282/s1, Figure S1: Photographs of the heat-set whey protein gels containing different CaCl_2_ concentrations; Figure S2: Phase angle of low-fat mayonnaises in the oscillatory shear test with the frequency sweep; Table S1: Average particle size (D4,3) of whey protein microparticles after homogenization at different speeds.
