# Supplementary material for: Role of Flaxseed Gum and Whey Protein Microparticles in Formulating Low-Fat Model Mayonnaises
Source: Foods. 2022 Jan 21;11(3):282. doi: 10.3390/foods11030282 (PMC8834398; doi:10.3390/foods11030282)
Supplement: Supplementary file 1 [file foods-11-00282-s001.zip › foods-1501921-supplementary.pdf]

Table S1 Average particle size ($D_{4,3}$) of whey protein microparticles after homogenization at different speeds

Speed (rpm)	$D_{4,3}$ (μm)	
	10 mM $CaCl_2$	15 mM $CaCl_2$
9000	40.01 ± 0.79 [a]	110.13 ± 0.05 [a]
12000	36.17 ± 0.61 [b]	78.64 ± 0.37 [b]
15000	16.28 ± 0.18 [c]	57.69 ± 0.20 [c]
18000	15.15 ± 0.11 [d]	54.10 ± 0.32 [d]
21000	9.94 ± 0.21 [e]	32.39 ± 0.78 [e]

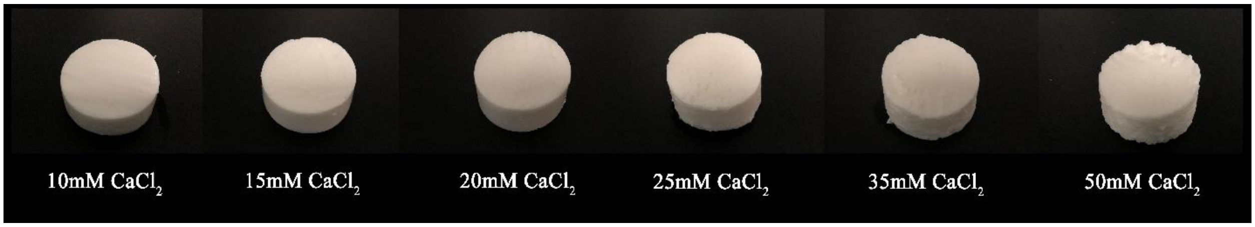

Figure S1 Photographs of the heat-set whey protein gels containing different $CaCl_2$ concentrations.

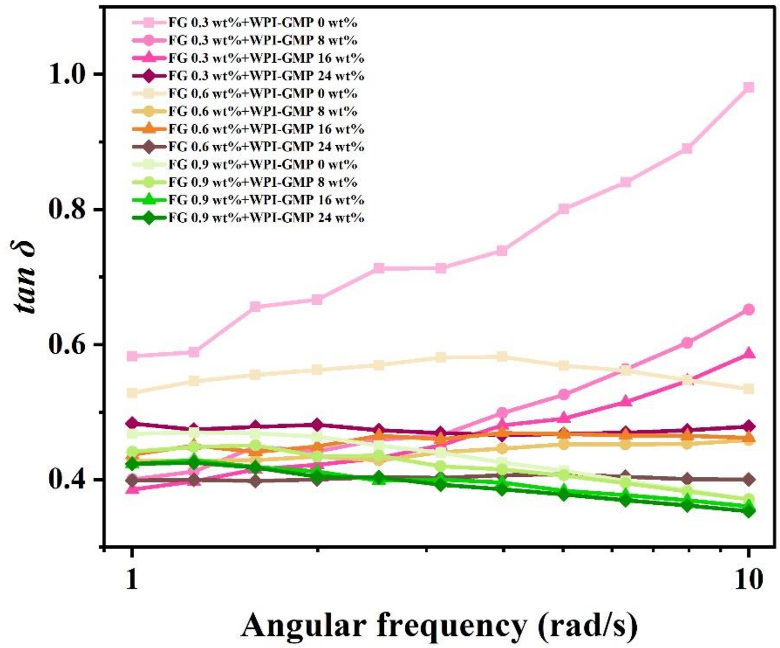

Figure S2 Phase angle of low-fat mayonnaises in the oscillatory shear test with the frequency sweep.
